# Dietary Microplastic Exposure in Athletes: Implications for Metabolism, Gut Health, and Performance

**DOI:** 10.3390/nu18142398

**Published:** 2026-07-22

**Authors:** Rosaria Meccariello, Maria Giovanna Tafuri, Stefania D’Angelo

**Affiliations:** 1 Department of Medical, Human Movement, and Well-Being Sciences (DiSMMeB), Parthenope University of Naples, 80133 Naples, Italy; rosaria.meccariello@uniparthenope.it; 2 Department of Literary, Pegaso Telematic University, 80143 Naples, Italy; mariagiovanna.tafuri@unipegaso.it

**Keywords:** microplastics, nanoplastics, dietary exposure, sports nutrition, athletes, gut microbiota, oxidative stress, mitochondrial dysfunction, exercise performance

## Abstract

Microplastics (MPs) are emerging environmental contaminants increasingly detected in foods, beverages, and food-contact materials, making dietary intake a relevant route of human exposure. In sports nutrition, this issue may be particularly important because athletes often have high food and fluid consumption, frequent use of packaged sports nutrition products, dietary supplements, bottled beverages, and sport-specific hydration strategies. This narrative review, supported by a structured literature search, examines dietary MP exposure and its potential relevance to gastrointestinal function, gut microbiota, oxidative stress, inflammation, mitochondrial activity, endocrine regulation, metabolism, recovery, adaptation, and performance-related outcomes in athletes. Current evidence suggests that MPs and nanoplastics may interact with biological systems through mechanisms involving intestinal barrier disruption, microbiota alterations, inflammatory activation, oxidative damage, mitochondrial perturbation, endocrine-disrupting chemicals, and metabolic dysregulation. However, most available data derive from in vitro studies, animal models, food contamination analyses, exposure-estimation studies, and indirect human biomonitoring evidence. Direct studies in athletic populations are currently lacking. Therefore, the possible implications of MP exposure on recovery, adaptation, and exercise performance should be interpreted as biologically plausible but unproven. From a practical perspective, evidence-informed strategies may include reducing avoidable plastic-related exposure while maintaining adequate hydration, energy availability, nutrient timing, supplement quality, and dietary patterns that support antioxidant defenses, inflammatory balance, gut health, and physiological resilience. Future research should prioritize standardized exposure assessment, validated biomarkers, human biomonitoring, and sport-specific studies evaluating MP exposure in relation to physiological and performance-related outcomes.

## 1. Introduction

Microplastics (MPs), commonly defined as plastic particles smaller than 5 mm, have emerged as pervasive environmental contaminants distributed across marine, terrestrial, and atmospheric systems [1,2]. Owing to their persistence, resistance to degradation, and continuous environmental release, MPs can accumulate in ecosystems and enter the human food chain. Human exposure is therefore increasingly considered unavoidable and may occur through ingestion, inhalation, and, to a lesser extent, dermal contact [3,4,5,6].

Dietary intake is one of the most consistent and biologically relevant routes of chronic MP exposure. MPs have been detected in several food and beverage matrices, including seafood, salt, honey, drinking water, bottled beverages, and processed foods [2,7,8,9,10]. Current exposure estimates suggest that humans may ingest tens of thousands of plastic particles annually through food and beverages, although these estimates remain highly uncertain because of differences in analytical methods, particle-size detection limits, food matrices, and exposure assumptions [2,4,10,11]. Thus, available data support the relevance of dietary exposure, but they do not yet provide precise individual-level estimates of MP intake.

Previous reviews have extensively addressed MPs and nanoplastics as environmental and human-health contaminants, focusing on their sources, environmental transport, ecosystem effects, exposure routes, toxicological mechanisms, analytical uncertainties, and potential health implications in the general population [3,4,5,11,12,13]. Other reviews have specifically examined MPs and nanoplastics in agri-food systems, including soil contamination, plant uptake, translocation to edible tissues, food safety, and dietary exposure [14]. In addition, organ-specific and cardiovascular-focused reviews have highlighted gastrointestinal, respiratory, cardiovascular, reproductive, immune, and nervous-system concerns, including vascular inflammation, endothelial dysfunction, oxidative stress, and immune activation as plausible pathways linking micro- and nanoplastic exposure to human health outcomes [15,16]. However, these reviews have generally focused on environmental systems, food safety, general human health, or specific disease-related outcomes, whereas the relevance of dietary MP exposure to athletes and sports nutrition remains largely unexplored.

**Figure 1:**
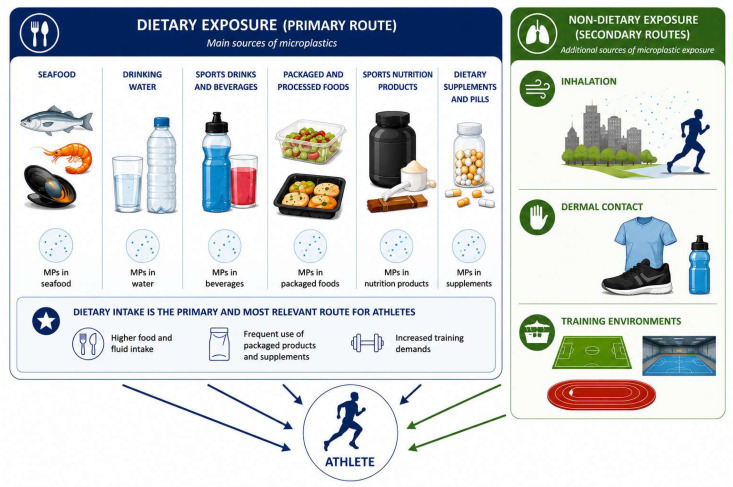
Exposure pathways of microplastics relevant to athletes. Dietary exposure is the primary route considered in this review and may occur through seafood, drinking water, bottled beverages, sports drinks, packaged foods, sports nutrition products, dietary supplements, and food-contact materials. Non-dietary routes, including inhalation of airborne particles in urban, indoor, and sport-specific environments, dermal contact with synthetic textiles or equipment, and exposure from synthetic training surfaces, may also contribute to overall exposure. The figure illustrates potential exposure pathways rather than quantified athlete-specific risk.

This gap is important because athletes may present distinctive exposure scenarios and physiological demands. High energy expenditure, increased food frequency, elevated fluid consumption, frequent use of packaged foods and beverages, sports drinks, protein powders, energy bars, and dietary supplements may increase opportunities for dietary contact with plastic particles. In addition, some sport-related dietary patterns, including high-protein, seafood-rich, or convenience-based diets during training, competition, and travel, may further contribute to potential MP exposure through food-chain transfer and food-contact materials [2,9,14]. In athletes, dietary exposure may therefore be particularly relevant because of elevated food and fluid intake, frequent use of packaged sports nutrition products, supplements, and sport-specific hydration practices (Figure 1).

Non-dietary routes may also be relevant in athletic settings. Airborne MPs may be inhaled during training, particularly in urban environments, indoor sports facilities, and synthetic turf fields, where material abrasion and resuspension may increase particle dispersion [3,6,17]. Dermal exposure may occur through contact with synthetic textiles, sports equipment, and contaminated environmental surfaces, although transdermal absorption remains poorly characterized and is likely to be limited compared with ingestion and inhalation [11,18]. Nevertheless, because this review focuses on sports nutrition, dietary exposure is considered the primary route of interest.

Beyond exposure, exercise-related physiology may influence the interpretation of potential MP effects. Athletes are characterized by high metabolic flux, repeated oxidative and inflammatory challenges, substantial gastrointestinal demands, and tightly regulated recovery and adaptation processes. Experimental and human-health reviews suggest that MPs and nanoplastics may interact with biological systems through oxidative stress, inflammatory signaling, gut barrier dysfunction, microbiota alterations, mitochondrial perturbation, endocrine or metabolic pathways, and vascular responses [5,13,15,16,19,20,21,22]. However, most of this evidence derives from in vitro studies, animal models, or indirect human observations. Whether these mechanisms translate into measurable effects on recovery, adaptation, or performance in athletes remains unknown.

Despite growing interest in the environmental and toxicological dimensions of MP exposure, direct evidence in athletic populations is almost absent. This represents an important gap, particularly because nutrition plays a central role in training adaptation, gastrointestinal tolerance, metabolic regulation, immune function, vascular health, and recovery [17,23,24]. Therefore, the relevance of MPs in sports nutrition should currently be interpreted as a plausible but unproven research question, rather than as an established determinant of athletic performance.

The aim of this narrative review is to provide a nutrition-centered and athlete-oriented analysis of MP exposure. Specifically, the review examines: (i) dietary and sport-related sources of MP exposure; (ii) gastrointestinal fate, absorption, and potential interactions with the gut microbiota; (iii) biochemical and metabolic mechanisms suggested by experimental and human evidence; and (iv) possible implications for recovery, adaptation, and exercise-related outcomes. By integrating evidence from environmental science, food safety, toxicology, human biomonitoring, cardiovascular research, and sports nutrition, this review proposes a conceptual framework for future research. The conclusions should be interpreted as evidence-informed and hypothesis-generating, rather than as causal or performance-specific recommendations.

## 2. Methods

This article was designed as a narrative review supported by a structured literature search to synthesize current evidence on dietary microplastic exposure and its potential implications for gut health, metabolism, physiological resilience, and exercise-related outcomes in athletes.

Relevant literature was identified through searches of PubMed, Scopus, and Web of Science, supplemented by Google Scholar. The literature search covered publications from database inception to July 2026 and was limited to articles published in English. Search terms were used alone and in combination and included: “microplastics”, “nanoplastics”, “micro- and nanoplastics”, “dietary exposure”, “food contamination”, “food packaging”, “bottled water”, “human exposure”, “human biomonitoring”, “gut microbiota”, “intestinal permeability”, “intestinal barrier”, “oxidative stress”, “inflammation”, “mitochondrial dysfunction”, “endocrine disruption”, “metabolism”, “sports nutrition”, “athletes”, “exercise”, “recovery”, and “performance”. Reference lists of relevant original articles, systematic reviews, narrative reviews, and authoritative reports were also manually screened to identify additional sources.

Peer-reviewed original studies, systematic and narrative reviews, experimental studies, human biomonitoring studies, and authoritative reports addressing microplastic exposure, biological mechanisms, food safety, dietary intake, or health-related outcomes were considered eligible. Because direct evidence in athletes is currently very limited, evidence from general human populations, animal models, and in vitro studies was included when mechanistically relevant to gastrointestinal, metabolic, inflammatory, endocrine, mitochondrial, or exercise-related pathways. Studies were excluded when they focused exclusively on environmental occurrence without biological, toxicological, dietary, or health-related implications.

Findings were synthesized qualitatively and organized into thematic sections covering exposure pathways, gastrointestinal fate and absorption, gut microbiota interactions, oxidative and inflammatory mechanisms, endocrine and metabolic pathways, performance-related implications, and nutritional strategies. Throughout the review, evidence was interpreted according to its source, distinguishing among in vitro findings, animal studies, indirect human evidence, human biomonitoring data, and the absence of athlete-specific evidence. Given the narrative scope of the review and the heterogeneity of study designs, exposure metrics, biological models, and outcomes, no formal meta-analysis was performed.

This review was not intended to provide an exhaustive quantitative synthesis of all available records and did not follow a formal systematic review protocol. Therefore, no PRISMA flow diagram, formal risk-of-bias assessment, or meta-analytic synthesis was conducted. The interpretation of the evidence emphasizes biological plausibility, evidence mapping, and hypothesis generation rather than causal inference or performance-specific recommendations.

## 3. Dietary Exposure in Athletes

### 3.1. Main Dietary Sources of Microplastic Exposure

Dietary intake is increasingly recognized as one of the most relevant routes of human exposure to microplastics, as food and beverages represent repeated and unavoidable sources of contact throughout life [2,3,4,8,25]. MPs have been detected in a wide range of dietary items, including seafood, bottled water, table salt, honey, sugar, fruits, vegetables, and processed foods [2,14,26,27]. However, current estimates of dietary MP intake remain highly variable because they depend on the food matrix analyzed, particle-size range, analytical method, contamination-control procedures, and assumptions used to extrapolate intake [2,4,10,11,12]. The evidence discussed in this section mainly derives from analyses of contaminated food and beverage matrices and from exposure-estimation studies; direct in vivo assessments of dietary MP intake in athletes are currently lacking. Therefore, the relevance of dietary MP exposure in athletes should be interpreted as a plausible exposure scenario rather than as a quantitatively established risk.

In athletes, this issue may be particularly relevant because nutritional practices are typically characterized by higher total food intake, increased fluid consumption, and frequent use of packaged sports nutrition products. These factors may create a distinctive exposure scenario compared with the general population. However, direct measurements of dietary MP exposure in athlete-specific cohorts are currently lacking; therefore, any assumption of higher exposure remains plausible but unconfirmed.

### 3.2. Hydration Practices and Packaged Beverages

Fluid consumption is a major potential contributor to dietary MP exposure in athletes. Adequate hydration is essential for thermoregulation, cardiovascular stability, metabolic efficiency, and exercise performance. Depending on training load, environmental conditions, and sport discipline, athletes may consume several liters of water or beverages daily. Bottled water has consistently been identified as a source of MP exposure, with measurable contamination reported across different brands and packaging types [26,27,28]. This contamination may originate from both source water and packaging materials, including polyethylene terephthalate bottles and polypropylene caps [27]. For athletes who rely on bottled water during training, travel, and competition, repeated intake of packaged beverages may represent a consistent route of exposure. This may be particularly relevant in endurance disciplines, where fluid intake is chronically elevated and may involve repeated use of plastic bottles or squeeze bottles exposed to mechanical stress, heat, and repeated handling. Nevertheless, the extent to which these practices increase internal MP burden has not yet been directly evaluated in athletes.

Sports drinks, recovery beverages, and energy drinks may represent additional sources of exposure. These products are commonly packaged in plastic containers or multilayer materials and may undergo industrial processing, storage, and transport conditions that could contribute to particle contamination. MPs have also been detected in soft drinks and related beverages, suggesting that contamination is not limited to water alone [28,29,30,31]. Within sports nutrition contexts, this is relevant because carbohydrate–electrolyte drinks, recovery shakes, and protein-based beverages are often consumed before, during, and after exercise. Therefore, exposure may be not only repeated but also temporally concentrated around exercise sessions, when gastrointestinal permeability and physiological stress may already be increased. This possible interaction remains hypothetical and requires direct investigation.

### 3.3. Sports Nutrition Products, Supplements, and Packaging

Processed foods and convenience products may further contribute to dietary MP exposure. Athletes frequently consume ready-to-eat meals, packaged snacks, energy bars, gels, meal replacements, protein powders, carbohydrate supplements, and recovery products to meet elevated nutritional demands and accommodate intensive training schedules.

These products may encounter plastic materials during manufacturing, packaging, transport, storage, and preparation. Mechanical abrasion, repeated opening and closing of containers, prolonged storage, and mixing or shaking procedures may increase the likelihood of particle release and contamination [31,32]. Protein powders and carbohydrate supplements may be particularly relevant because they are often stored in plastic containers and repeatedly handled, scooped, mixed, or consumed using plastic utensils, shaker bottles, and sports bottles.

However, direct quantity of MPs in sports supplements remains limited. Therefore, their relevance should be interpreted as a plausible exposure pathway based on packaging characteristics and patterns of use, rather than as a demonstrated major source of MP intake in athletes.

### 3.4. Food Choices, Seafood, and Plant-Derived Foods

Food choice is another important determinant of dietary MP exposure in athletes. Many athletes follow high-energy diets with substantial contributions from animal-derived protein sources, including fish and seafood, which are among the most frequently reported dietary reservoirs of MPs [2,9,33]. Marine organisms may ingest MPs from contaminated environments, resulting in trophic transfer along the food chain [9,14]. Shellfish may be particularly relevant because they are often consumed whole, increasing the likelihood of ingesting particles retained in digestive tissues.

In endurance sports such as swimming, triathlon, and rowing, seafood may be included as part of a high-quality recovery diet due to its protein content, micronutrients, and omega-3 fatty acids. However, this nutritionally beneficial dietary pattern may also contribute to dietary MP exposure. Importantly, this should not be interpreted as a reason to discourage seafood intake, but rather as a sign that sports nutrition requires a balanced risk–benefit perspective.

Plant-derived foods may also contribute to dietary exposure, as MPs and nanoplastics have been reported in fruits, vegetables, spices, and other plant-based products [2,14,26,27]. This is relevant because athletes are often encouraged to consume high amounts of fruits, vegetables, whole grains, and other plant-based foods to support antioxidant intake, gut health, and recovery. As with seafood, the nutritional value of these foods should not be overlooked. Rather, the presence of MPs in plant-derived foods highlights the broader challenge of food-chain contamination and the need for improved monitoring and standardized analytical methods.

### 3.5. Cumulative Exposure and Athlete-Specific Uncertainty

Food processing and packaging conditions may influence both the quantity and characteristics of MPs present in foods. Thermal stress, ultraviolet exposure, repeated compression, mechanical abrasion, and long-term storage may promote fragmentation of plastic materials and particle migration into edible products [8,31].

This issue may be particularly relevant in sports settings, where foods and beverages are often transported in warm environments, stored in training bags or vehicles, and consumed from single-use or repeatedly used plastic containers. Reused shaker bottles, squeeze bottles, and food containers may also be potential sources of exposure because of repeated wear, washing, and mechanical degradation.

Total dietary volume is an added factor that may contribute to cumulative exposure. Even when MP concentrations in individual food items are low, total intake in athletes may become relevant because of the high quantity of food and fluids consumed. During periods of intensified training or competition, athletes often increase caloric intake through multiple meals, snacks, supplements, and beverages distributed throughout the day. In this context, exposure is unlikely to arise from a single dominant source, but rather from repeated contact with multiple low-level sources.

This cumulative exposure model may better reflect real-world sports nutrition practices than isolated contamination estimates [4,11]. Current evidence is limited by the lack of athlete-specific dietary exposure assessments, biomonitoring studies, and standardized methods for evaluating MP intake in physically active populations [4,11,12]. Most available estimates are based on consumption patterns derived from the general population rather than sport-specific nutritional models [2,10]. Consequently, the actual exposure burden in athletes remains uncertain. Future research should investigate discipline-specific dietary patterns, compare packaged and minimally packaged strategies, and decide whether periods of intensified training, competition, or travel are associated with increased internal MP burden.

Overall, available evidence supports the hypothesis that athletes may present a distinctive dietary exposure profile. Elevated fluid intake, frequent consumption of sports beverages and supplements, reliance on packaged foods, seafood-rich dietary patterns, and repeated use of plastic food-contact materials may collectively contribute to cumulative exposure. However, the quantitative magnitude and physiological relevance of this burden remain to be proved [2,4,10,11,27,29,31,33].

## 4. Gastrointestinal Fate, Absorption, and Human Biomonitoring of Microplastics

### 4.1. Gastrointestinal Interactions and Determinants of Particle Fate

Following dietary ingestion, MPs interact directly with the gastrointestinal (GI) tract, which represents the primary interface between environmental exposure and potential biological responses. Although this review focuses mainly on MPs, evidence on nanoplastics is also considered when relevant, because particle size is a critical determinant of intestinal interaction and possible translocation. The fate of MPs within the GI system depends on several physicochemical characteristics, including particle size, shape, surface charge, polymer composition, surface aging, and the presence of adsorbed contaminants or biological molecules [5,8,11,12,25]. These properties influence not only particle retention and fecal elimination, but also local interactions with the intestinal mucus layer, epithelial cells, immune structures, digestive fluids, and luminal microbiota.

Larger MP particles, particularly those above approximately 150 μm, are generally considered unlikely to cross the intestinal barrier and are expected to be predominantly eliminated through feces [4,8,12]. In contrast, smaller MPs and nanoplastics, especially particles in the submicrometric and nanometric range, may have greater potential to interact with epithelial surfaces and, under specific experimental conditions, cross biological barriers [5,11,13,34,35]. Proposed uptake mechanisms include endocytosis by enterocytes, transcytosis through microfold cells in Peyer’s patches, uptake by phagocytic immune cells, and paracellular passage through tight junctions when barrier integrity is compromised [12,13,15,33].

However, these mechanisms should be interpreted with caution. Most evidence supporting intestinal uptake and translocation derives from in vitro epithelial models, animal studies, and controlled experimental exposures rather than from direct human dietary studies. In vitro models, including Caco-2 monolayers and other intestinal epithelial systems, have shown that small plastic particles can interact with epithelial cells, alter barrier properties, and, in some cases, cross cellular or paracellular barriers [5,12,15]. Nevertheless, these models only partially reproduce the complexity of the human intestinal environment, including mucus, immune cells, microbiota, digestive secretions, peristalsis, and realistic dietary matrices.

### 4.2. Experimental Evidence for Absorption and Systemic Translocation

Experimental in vivo evidence comes mainly from animal models, particularly rodents, fish, and other aquatic organisms exposed to defined plastic particles, often polystyrene MPs or nanoplastics [5,15,34,35]. In these studies, particles have been detected primarily in the GI tract and, in some cases, in extra-intestinal tissues such as the liver, spleen, lymphatic tissues, kidney, reproductive organs, and brain [5,12,13,15,34,35]. These findings suggest that a fraction of ingested particles, particularly smaller particles and nanoplastics, may overcome intestinal barriers and reach systemic compartments.

The methods used in experimental studies vary considerably. Many in vitro and animal studies have used fluorescently labelled plastic particles, followed by fluorescence microscopy, confocal microscopy, histological analysis, or tissue imaging to assess uptake and distribution [5,12]. Other studies have used spectroscopic or polymer-identification techniques to confirm the presence of plastic particles in biological matrices. While these approaches are useful for mechanistic investigation, they have important limitations. Fluorescent particle studies may be affected by dye leaching, particle aggregation, signal misinterpretation, or unrealistic exposure concentrations. Similarly, high-dose animal studies may not reflect chronic low-dose human dietary exposure.

Therefore, although experimental studies support the biological plausibility of intestinal uptake and systemic translocation, they do not yet define the real magnitude, kinetics, or dose–response relationships of MP absorption in humans. Importantly, no study has directly quantified MP absorption, tissue distribution, or elimination in athletes.

### 4.3. Human Biomonitoring: Biological Matrices, Methods, and Analytical Limitations

Human evidence remains more limited and is primarily observational. MPs and nanoplastics have been reported in several biological matrices, including feces, blood, lung tissue, placenta, liver tissue, urine, semen, atherosclerotic plaques, brain tissue, and, more recently, skeletal tissues [16,36,37,38,39,40,41,42,43]. These findings indicate that plastic particles can be detected beyond external environmental matrices and may reach different human biological compartments. However, detection in human tissues should not be interpreted as proof of tissue-specific toxicity, functional impairment, causal involvement in disease, or relevance to athletic performance.

Current methods for detecting MPs in human samples include visual microscopy combined with spectroscopic techniques such as micro-Fourier transform infrared spectroscopy and Raman spectroscopy, as well as thermal and mass spectrometry-based approaches such as pyrolysis–gas chromatography–mass spectrometry [4,11,12,44]. Spectroscopic techniques can provide information on particle size, shape, and polymer type, but their sensitivity decreases for very small particles and nanoplastics. Thermal and mass spectrometry-based methods can improve polymer identification and quantification, but they usually provide less information on particle morphology, localization, and tissue distribution.

Across human biomonitoring studies, major methodological limitations remain. These include possible contamination during sampling and laboratory processing, heterogeneous digestion and extraction protocols, lack of harmonized quality-control procedures, different lower size-detection limits, variable reporting units, and difficulty distinguishing external contamination from truly internalized particles [4,11,12,44,45]. These limitations are particularly relevant when comparing studies across biological matrices or attempting to infer exposure burden, tissue accumulation, or health effects.

Human biomonitoring studies therefore demonstrate detectability, but they do not yet establish absorption efficiency, systemic distribution kinetics, dose–response relationships, or clinical consequences. This distinction is essential in the context of sports nutrition, where no athlete-specific biomonitoring data are currently available.

### 4.4. Local Gastrointestinal Effects and Interaction with Co-Contaminants

Beyond systemic uptake, MPs may exert local effects within the GI tract. Physical interactions between particles and the intestinal mucosa may contribute to epithelial irritation, altered mucus secretion, and disruption of barrier integrity in experimental models [5,12,13,46]. These effects may facilitate increased intestinal permeability and translocation of luminal components, including bacterial products such as lipopolysaccharide. However, direct confirmation of these processes in humans remains limited.

Another important aspect of GI fate involves interactions between MPs and co-ingested chemical substances. MPs may act as carriers for environmental contaminants, including heavy metals, persistent organic pollutants, and plastic additives such as bisphenols and phthalates [3,5,9,11,13,47,48]. These compounds may adsorb onto particle surfaces and may do so after being released under gastrointestinal conditions, depending on pH, digestive enzymes, bile salts, polymer type, particle size, and surface aging. This “carrier effect” may contribute to combined particle- and chemical-mediated biological responses, although its relevance under realistic human dietary exposure remains uncertain.

The gastrointestinal environment may also modify the physicochemical identity of MPs. Exposure to gastric acidity, intestinal enzymes, bile salts, and dietary components may alter surface properties, aggregation behavior, and biomolecular adsorption [11,12,25]. In biological fluids, MPs may get a “protein corona”, namely a layer of adsorbed proteins and other biomolecules that can modify particle recognition, cellular uptake, and immune interactions [13,49]. These processes may influence biological responses, but their magnitude and relevance in humans are still insufficiently characterized.

### 4.5. Relevance to Athletes

In athletes, the gastrointestinal fate of MPs should be interpreted within the context of exercise-induced physiological changes. Prolonged or high-intensity exercise can redistribute blood flow away from the splanchnic region toward working muscles, leading to relative intestinal hypoperfusion, epithelial stress, increased oxidative burden, and transient increases in intestinal permeability, commonly described as exercise-induced gastrointestinal syndrome [48]. In this context, MPs present in the intestinal lumen during or around exercise could theoretically interact with a more vulnerable epithelial barrier.

However, this remains a hypothesis. No study has directly tested whether exercise modifies MP retention, epithelial uptake, systemic translocation, or elimination. Similarly, no study has examined whether MP exposure affects GI symptoms, nutrient absorption, recovery, or performance in athletes. Therefore, the possible interaction between MP exposure and exercise-induced intestinal stress should be considered mechanistically plausible but not demonstrated.

Frequent ingestion of fluids, sports drinks, gels, protein shakes, and supplements during training or competition may introduce MPs into the GI tract at times when gastrointestinal physiology is already altered. This temporal overlap may be relevant for future research, particularly in endurance and ultra-endurance sports, where GI symptoms and barrier stress are common. Nevertheless, the current evidence does not allow conclusions regarding clinically meaningful effects in athletes.

### 4.6. Remaining Uncertainties and Research Priorities

Significant uncertainties remain regarding the absorption, distribution, metabolism, and elimination of MPs in humans. Current knowledge is derived primarily from in vitro studies, animal models, food occurrence studies, and observational biomonitoring data, while direct human evidence remains limited and athlete-specific evidence is absent [4,5,11,12,15,34,35]. Variability in particle characteristics, exposure conditions, analytical methods, and biological matrices complicates comparisons across studies and limits the interpretation of existing findings.

Future research should quantify MP absorption under realistic dietary conditions, identify reliable biomarkers of internal exposure, harmonize analytical methods, and investigate whether exercise modifies intestinal uptake, systemic distribution, or elimination. In athletes, studies should integrate dietary exposure assessment with biological matrices, gut permeability markers, inflammatory and oxidative biomarkers, GI symptoms, and recovery-related outcomes.

Overall, the GI tract is a critical interface where dietary MPs may interact with biological systems. Experimental evidence supports possible local epithelial effects, microbiota interactions, and limited systemic translocation, especially for smaller particles and nanoplastics. However, the quantitative magnitude and physiological relevance of these processes in humans stay uncertain. In athletes, the combination of repeated dietary exposure, exercise-related intestinal stress, and high metabolic demands creates a relevant research context, but current conclusions must remain provisional and hypothesis-generating rather than causal or performance-specific.

## 5. Microplastics–Microbiota Axis

### 5.1. Gut Microbiota Is a Biological Target of Microplastic Exposure

The gut microbiota plays a fundamental role in human health by contributing to nutrient metabolism, immune regulation, intestinal barrier integrity, short-chain fatty acid (SCFA) production, and systemic physiological homeostasis [50,51]. Increasing evidence suggests that the gut microbial ecosystem is sensitive to environmental contaminants, including microplastics and nanoplastics [12,19,47,52,53]. The interaction between MPs and the gut microbiota, often referred to as the “microplastics–microbiota axis”, is a potential pathway through which dietary exposure may influence gastrointestinal, immune, and metabolic responses [12,47,52,54].

However, the current evidence base remains predominantly preclinical. Most data derive from animal models, especially rodents and aquatic organisms, or from mechanistic reviews integrating in vitro and in vivo findings [12,19,50,53,55]. Direct human studies linking dietary MP exposure to gut microbiota composition are scarce, and athlete-specific studies are currently lacking. Therefore, the microplastics–microbiota axis should be interpreted as a mechanistically plausible pathway rather than an established determinant of gut health or performance in athletes.

### 5.2. Experimental Evidence from Animal and In Vitro Models

Experimental evidence, mainly from in vivo animal models, suggests that MP exposure may alter both gut microbiota composition and microbial diversity [19,50,53,55]. In a rodent model, Lu et al. exposed male ICR mice to polystyrene MPs of different sizes for five weeks and reported changes in gut microbial composition together with alterations in hepatic lipid metabolism [50]. These findings support a possible gut–liver metabolic link, but the controlled exposure conditions, particle characteristics, and laboratory setting limit direct translation to chronic human dietary exposure.

In zebrafish, Qiao et al. exposed animals to 5-μm polystyrene MPs for 21 days and observed intestinal inflammation, oxidative stress, and changes in both gut microbiome and metabolomic profiles [55]. These findings suggest that MP exposure may affect intestinal homeostasis and microbial metabolic activity in aquatic vertebrate models. Nevertheless, zebrafish studies are primarily useful for mechanistic insight and cannot be directly extrapolated to human athletes.

Across experimental models, reported microbiota alterations include reduced microbial diversity, shifts in the relative abundance of major bacterial groups such as *Firmicutes* and *Bacteroidetes*/*Bacteroidota*, and changes in taxa involved in immune regulation, lipid metabolism, epithelial homeostasis, and inflammatory signaling [50,52,53,55,56]. Some in vitro systems have also suggested that MPs may interact with epithelial and microbial components, but these models only partially reproduce the complexity of the intestinal lumen, including mucus, diet-derived substrates, bile acids, immune cells, microbial cross-feeding, and host metabolism.

### 5.3. Functional Consequences: Microbial Metabolites, SCFAs, and Barrier Regulation

A major potential consequence of microbiota disruption is altered microbial metabolic activity. The gut microbiota contributes to the production of SCFAs, including acetate, propionate, and butyrate, which are important for maintaining intestinal epithelial integrity, regulating immune responses, and supporting host energy metabolism [57,58,59]. Experimental studies and mechanistic reviews suggest that MP-induced dysbiosis may be associated with changes in SCFA-producing bacteria and reduced SCFA availability [19,47,53,56]. This mechanism may be relevant for athletes because SCFAs have been implicated in energy homeostasis, gut barrier function, immune regulation, and exercise-related metabolic adaptation [59]. However, direct evidence that MP exposure reduces SCFA production in athletes or impairs exercise recovery through SCFA-dependent pathways is currently unavailable. Therefore, this link should be considered indirect and hypothesis-generating. Microbial metabolites may also influence systemic inflammation, insulin sensitivity, lipid metabolism, and gut–muscle communication. In this context, MP-related microbiota disruption could theoretically affect metabolic resilience during training. Nevertheless, the available evidence does not yet establish whether the magnitude of microbiota changes observed in experimental models is sufficient to produce clinical or performance-relevant effects in humans.

### 5.4. Plastisphere, Co-Contaminants, and Epithelial–Immune Interactions

In addition to compositional changes, MPs may interact with the microbiota through direct physical and chemical mechanisms. Plastic particles can provide surfaces for microbial colonization, forming biofilm-like communities often referred to as the “plastisphere” [3,53,60]. These microbial assemblages may differ from native gut communities and, depending on environmental and host conditions, may include opportunistic or potentially pathogenic taxa. However, most plastisphere evidence comes from environmental and aquatic studies; its relevance within the human intestinal lumen remains insufficiently characterized.

MPs may also adsorb environmental contaminants, plastic additives, or other xenobiotics that can be released under gastrointestinal conditions [11,13,61,62]. These substances could exert selective antimicrobial pressure or alter microbial metabolism, thereby contributing to dysbiosis. At present, the biological relevance of this carrier effect under realistic dietary exposure remains uncertain, especially in humans.

Another relevant mechanism involves the interplay among MPs, the intestinal epithelium, gut microbiota, and the immune system. MP-induced dysbiosis may contribute to increased intestinal permeability and facilitate the translocation of microbial products, including lipopolysaccharide (LPS), into systemic circulation [12,19,47,63]. This process could promote low-grade inflammatory signaling, which has been associated with metabolic dysregulation and impaired physiological function [9]. However, this sequence has not been directly demonstrated in humans exposed to dietary MPs, nor has it been tested in athletes.

### 5.5. Relevance to the Microbiota–Gut–Muscle Axis in Athletes

The microbiota–gut–muscle axis provides an additional framework for considering the potential relevance of MP-induced microbiota alterations in athletes. Emerging evidence suggests that the gut microbiota may influence muscle function and exercise adaptation through modulation of energy availability, inflammatory balance, amino acid metabolism, SCFA production, and immune signaling [59,64]. Disruption of this axis could theoretically affect muscle function, recovery kinetics, and tolerance to training stress.

Athletes may be particularly sensitive to gut microbiota perturbations because of the combined effects of diet, training load, gastrointestinal stress, travel, sleep disruption, and supplement use. Prolonged or high-intensity exercise can transiently alter gut permeability and may influence microbiota composition [64]. In this context, MP exposure could theoretically interact with exercise-induced intestinal stress, but this remains speculative. Importantly, current evidence does not show that MPs impair muscle function, delay recovery, or reduce performance through microbiota-mediated mechanisms in athletes. The available evidence supports only a plausible biological pathway that requires validation in well-designed human studies.

### 5.6. Microplastics–Microbiota Axis: Limitations and Future Directions 

Despite growing interest in the microplastics–microbiota axis, several limitations restrict interpretation. First, most evidence derives from animal models or in vitro systems, while human data remain sparse [12,19,53]. Second, experimental studies differ substantially in particle size, polymer type, particle shape, surface charge, exposure dose, exposure duration, and route of administration. Third, many studies use exposure levels that may not reflect chronic dietary exposure in humans. Fourth, microbiota outcomes vary across sequencing methods, bioinformatic pipelines, and taxonomic classification systems. These factors limit comparability across studies and make it difficult to define dose–response relationships.

Future research should integrate dietary exposure assessment with metagenomics, metabolomics, transcriptomics, intestinal permeability markers, inflammatory biomarkers, and standardized MP detection methods. In athletes, longitudinal studies should examine whether dietary MP exposure is associated with gut microbiota composition, SCFA profiles, GI symptoms, training load, recovery markers, and exercise-related outcomes. Intervention studies comparing highly packaged versus minimally packaged sports nutrition strategies could also help clarify whether modifiable dietary practices influence internal MP burden or microbiota-related endpoints. Overall, the microplastics–microbiota axis represents a plausible mechanistic link between dietary exposure and systemic physiological responses. Experimental evidence, mainly from animal models, indicates that MPs may alter gut microbial composition, intestinal inflammation, oxidative stress, and microbial metabolism [19,50,53,55]. However, human evidence remains limited, and athlete-specific evidence is currently absent. Therefore, the relevance of this axis for sports nutrition, recovery, and performance-related outcomes remains to be proven.

Because current evidence is heterogeneous and largely indirect, Table 1 summarizes representative experimental, human biomonitoring, and methodological evidence, highlighting the main findings, limitations, and translational relevance for athletic populations.

## 6. Biochemical and Metabolic Disruption

### 6.1. Overview of the Evidence Base

**Figure 2:**
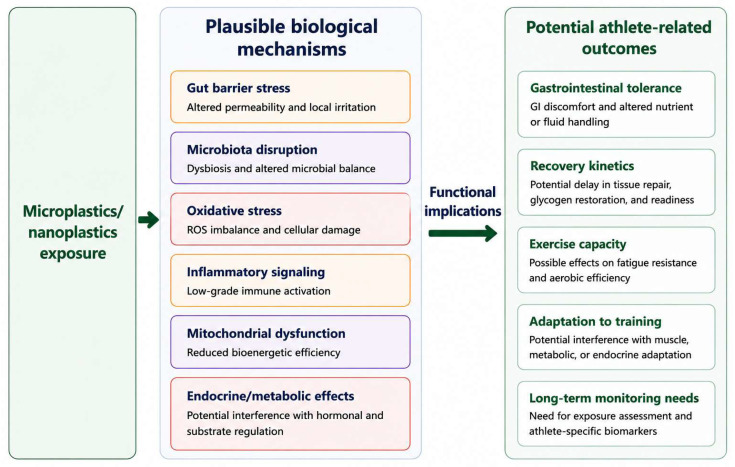
Plausible biological mechanisms linking microplastic/nanoplastic exposure to potential athlete-related outcomes. Microplastics and nanoplastics may interact with biological systems through interconnected mechanisms, including gut barrier stress, microbiota disruption, oxidative stress, inflammatory signaling, mitochondrial dysfunction, and endocrine/metabolic effects. These pathways may have functional implications for gastrointestinal tolerance, recovery kinetics, exercise capacity, adaptation to training, and long-term monitoring needs in athletes. However, the proposed outcomes should be interpreted as potential implications based mainly on mechanistic, in vitro, and animal evidence, while direct evidence in athletic populations is still lacking.

At the cellular level, experimental evidence suggests that MPs and nanoplastics may interfere with fundamental biochemical processes, primarily through oxidative stress, inflammatory signaling, mitochondrial perturbation, endocrine disruption, and altered metabolic regulation [5,12,13,15,65,66]. These mechanisms represent plausible links between environmental exposure and metabolic dysregulation and may be particularly relevant in athletes, whose performance and recovery depend on tightly regulated bioenergetic, redox, inflammatory, and endocrine processes [67,68] (Figure 2).

However, the current evidence base remains largely experimental. Most mechanistic findings derive from in vitro studies using human or mammalian cell lines, animal models exposed to defined plastic particles, and toxicological reviews integrating preclinical data [5,11,12,13]. Human evidence is mainly indirect or observational, and no study has directly evaluated whether MP exposure disrupts biochemical or metabolic pathways in athletes. Therefore, the mechanisms discussed in this section should be interpreted as biologically plausible but not yet established in sports nutrition or exercise-performance contexts.

### 6.2. Oxidative Stress and Cellular Damage

One of the most consistently reported responses to MP exposure in experimental models is increased generation of reactive oxygen species (ROS). MPs may induce oxidative stress through direct particle–cell interactions, lysosomal and mitochondrial stress, activation of immune signaling pathways, and release of associated chemical additives or adsorbed contaminants [65,66,67,68]. In vitro studies using epithelial, hepatic, renal, immune, and fibroblast-like cell models have reported reduced cell viability, increased ROS production, altered antioxidant enzyme activity, inflammatory marker expression, and, in some cases, genotoxicity after exposure to different polymer types and particle sizes [83,84,85].

Animal studies also support oxidative stress as a recurrent response to MP exposure. In rodent and aquatic models, MPs have been associated with altered antioxidant defenses, lipid peroxidation, tissue-specific oxidative injury, and changes in redox-sensitive signaling pathways [53,55,86]. However, these studies often use controlled exposure conditions, selected polymers such as polystyrene, and doses that may exceed typical human dietary exposure. Therefore, although oxidative stress is a coherent mechanism across experimental models, its magnitude and relevance under real-world human exposure remain uncertain.

Within exercise physiology, oxidative stress has a dual role. Moderate ROS production contributes to mitochondrial biogenesis, redox signaling, muscle adaptation, and training-induced remodeling, while excessive or sustained oxidative stress may impair contractile function, delay recovery, and contribute to fatigue [67,68]. In this context, MP-related oxidative stress could theoretically interfere with redox homeostasis in athletes. However, direct evidence that dietary MPs alter exercise-induced redox adaptation or recovery in athletic populations is currently lacking.

### 6.3. Inflammatory Signaling and Immune Activation

Inflammation is another potential pathway through which MPs may exert biological effects. Experimental studies suggest that MP exposure may activate inflammatory signaling cascades, including nuclear factor kappa B (NF-κB), and increase the production of pro-inflammatory mediators such as interleukin-6 (IL-6), tumor necrosis factor alpha (TNF-α), and interleukin-1β (IL-1β) [13,54,66,69,70]. These responses may occur through epithelial irritation, oxidative stress, activation of innate immune pathways, gut barrier disruption, or translocation of microbial products such as lipopolysaccharide.

In vitro studies have shown that MPs can induce inflammatory responses in different cellular systems, including epithelial and immune-related models [83,84,85]. Animal studies have also reported intestinal inflammation, altered cytokine profiles, and tissue-specific inflammatory responses following MP exposure [53,55,86]. Nevertheless, the translation of these findings to humans is limited by differences in exposure route, particle concentration, polymer type, exposure duration, and biological context. In athletes, inflammatory responses are tightly linked to training adaptation, tissue repair, immune function, and recovery. Acute inflammatory signaling is part of normal adaptation to exercise, whereas excessive or persistent low-grade inflammation may impair recovery and increase susceptibility to illness or overreaching [68]. Therefore, MP-induced inflammatory activation could theoretically influence recovery kinetics or adaptation to training. However, this is still speculative, as no human study has tested whether MP exposure changes inflammatory biomarkers in athletes.

### 6.4. Mitochondrial Dysfunction and Bioenergetic Regulation

Mitochondria are central to ATP production, substrate oxidation, redox balance, calcium handling, and skeletal muscle function. Experimental evidence suggests that MPs and nanoplastics may affect mitochondrial integrity and function, leading to altered mitochondrial membrane potential, impaired electron transport, increased ROS generation, and reduced ATP production [5,15,32,65,71,72,73]. These effects have been reported mainly in in vitro models and animal studies, where smaller particles and nanoplastics appear to have greater potential for intracellular interaction. The relevance of mitochondrial dysfunction is particularly important in sport and exercise physiology because endurance capacity, repeated-sprint ability, recovery, and metabolic flexibility depend on efficient mitochondrial function. Nevertheless, the available evidence does not demonstrate that MP exposure impairs mitochondrial bioenergetics in human skeletal muscle or reduces exercise capacity in athletes. At present, mitochondrial involvement should therefore be considered a mechanistic hypothesis derived from preclinical evidence rather than an established performance-related effect.

Future studies should investigate whether dietary MP exposure is associated with markers of mitochondrial function, oxidative phosphorylation, metabolic flexibility, or exercise-induced mitochondrial adaptation in humans. Such studies should also consider training status, diet quality, antioxidant intake, and exposure to other environmental stressors.

### 6.5. Endocrine and Metabolic Effects

In addition to direct particle-related effects, MPs may influence metabolic processes through their role as carriers of exogenous chemicals. MPs can adsorb environmental contaminants, including heavy metals, persistent organic pollutants, and endocrine-disrupting compounds such as bisphenols and phthalates [11,13,74,87]. These compounds may be released under gastrointestinal conditions and once absorbed, may interfere with hormonal pathways involved in energy balance, glucose homeostasis, lipid metabolism, reproductive function, and muscle protein regulation [43,74,75]. Emerging experimental evidence also suggests that MP exposure may influence lipid and glucose metabolism. In animal models, MP exposure has been associated with hepatic lipid accumulation, altered gut microbiota, insulin resistance, adiposity, and cardiometabolic alterations [50,76,86]. For example, experimental studies in mice have suggested that polystyrene MP exposure may contribute to metabolic disruption through dysbiosis, inflammation, and altered hepatic or adipose metabolic signaling [50,76]. However, these findings derive from animal models and cannot be directly extrapolated to human athletes.

From a sports nutrition perspective, glucose regulation, insulin sensitivity, lipid oxidation, endocrine status, and muscle protein turnover are key determinants of training adaptation and recovery. Therefore, endocrine or metabolic effects of MPs could be relevant if confirmed in humans. At present, however, there is no direct evidence that dietary MP exposure alters glucose metabolism, substrate use, hormonal adaptation, or muscle protein synthesis in athletes.

### 6.6. Interactions Among Oxidative Stress, Inflammation, and Metabolism

The interaction among oxidative stress, inflammation, mitochondrial dysfunction, and metabolic regulation may create a self-reinforcing cycle. Increased ROS production can activate inflammatory pathways, while inflammatory signaling may further amplify oxidative stress and impair mitochondrial function. Mitochondrial dysfunction, in turn, may alter energy metabolism and increase cellular vulnerability to additional stressors [5,12,13,15]. This integrated response may contribute to a sustained state of cellular or metabolic stress in experimental models. However, its relevance under realistic dietary exposure conditions is still uncertain. Human studies are needed to find whether environmental or dietary MP exposure is associated with measurable changes in oxidative, inflammatory, mitochondrial, or metabolic biomarkers, particularly in physically active populations.

### 6.7. Influence of Particle Characteristics and Biological Identity

The biological effects of MPs are strongly influenced by their physicochemical characteristics. Particle size, shape, polymer composition, surface charge, aging, crystallinity, and surface roughness can affect cellular interaction, uptake, reactivity, and potential toxicity [11,12,88,89]. Nanoplastics, because of their smaller size and larger surface-area-to-volume ratio, may show greater biological reactivity and higher potential for intracellular interaction than larger MPs. In biological fluids, MPs may get a biomolecular corona composed of proteins, lipids, metabolites, and other molecules adsorbed onto the particle surface [13,49,77]. This corona may alter the biological identity of MPs, influencing immune recognition, cellular uptake, biodistribution, and toxicological responses. However, the composition and functional consequences of MP coronas under realistic human dietary exposure conditions are still insufficiently characterized.

### 6.8. Relevance to Athletes and Future Directions

Despite increasing evidence from experimental models, significant uncertainties are still regarding the biochemical and metabolic effects of MPs in humans, particularly in athletic populations. Most available data derive from in vitro and animal studies, and their extrapolation to real-world human exposure scenarios is limited [4,5,11,12]. Furthermore, the interaction between MP exposure and exercise-induced physiological responses has not been extensively investigated.

In athletes, future research should focus on controlled observational and interventional studies assessing dietary MP exposure alongside biomarkers of oxidative stress, inflammation, gut barrier integrity, mitochondrial function, endocrine regulation, glucose metabolism, lipid metabolism, and recovery. Studies should also distinguish between acute exercise-related responses and chronic exposure-related changes, as both oxidative stress and inflammation are normal components of training adaptation.

Overall, MPs may influence key pathways involved in redox balance, inflammatory regulation, mitochondrial function, endocrine signaling, and metabolic homeostasis. However, current evidence is largely derived from preclinical studies and indirect human observations. The magnitude and functional relevance of these effects in athletes remain to be proven, and the findings should be interpreted as mechanistic plausibility rather than evidence of impaired performance or recovery.

## 7. Endocrine Disruption and Hormonal Implications

### 7.1. Microplastics as Carriers of Endocrine-Disrupting Chemicals

MPs should be considered not only as physical particles, but also as potential carriers of endocrine-disrupting chemicals (EDCs), including bisphenols, phthalates, flame retardants, plasticizers, and other plastic-associated additives [3,9,11,12,13,74,77]. These compounds may leach from plastic materials, adsorb onto MP surfaces from the surrounding environment, or be released under gastrointestinal conditions following ingestion [5,9,77]. Therefore, the endocrine relevance of MPs may derive from both particle-related effects and chemical-mediated effects associated with additives or adsorbed contaminants.

However, it is important to distinguish between these two mechanisms. Evidence that MPs can carry or release EDCs is relatively well supported by environmental and experimental studies, while evidence that dietary MPs cause clinically meaningful endocrine disruption in humans is still limited. In addition, most available studies do not clearly separate the effects of the plastic particle itself from those associated chemicals. This distinction is particularly important in the context of sports nutrition, where no athlete-specific data is currently available.

### 7.2. Potential Mechanisms of Endocrine Disruption

Endocrine disruption is a plausible mechanism through which MPs and MP-associated chemicals may influence metabolic regulation and physiological homeostasis. EDCs associated with plastics may interfere with nuclear hormone receptors, including estrogen receptors, androgen receptors, thyroid hormone receptors, peroxisome proliferator-activated receptors, and glucocorticoid-related signaling pathways [12,43,74,77]. These interactions may alter gene expression, cellular differentiation, reproductive signaling, adipogenesis, glucose metabolism, lipid metabolism, and energy balance.

Experimental evidence suggests that MP exposure may also interact with endocrine and metabolic pathways indirectly through oxidative stress, inflammation, mitochondrial dysfunction, gut barrier disruption, and microbiota alterations [5,12,13,15]. These mechanisms may converge on hormonal regulation because endocrine function is closely linked to inflammatory tone, metabolic status, redox balance, and gut-derived signaling. Nevertheless, most of this evidence derives from in vitro systems and animal models, and its translation to human physiology is still uncertain.

Alterations in insulin signaling, glucose homeostasis, lipid metabolism, thyroid-related pathways, and reproductive hormones have been reported in experimental studies of MPs or plastic-associated chemicals [5,15,43,74,77]. However, direct human evidence is still sparse, and causality cannot currently be inferred.

### 7.3. Hormonal Regulation in Athletes

Within the context of sports nutrition, hormonal regulation plays a central role in performance, recovery, and adaptation to training. Anabolic hormones and growth-related mediators, including testosterone, growth hormone, insulin, and insulin-like growth factor 1, support muscle protein synthesis, glycogen resynthesis, tissue repair, and adaptive remodeling. Catabolic and stress-related hormones, such as cortisol and catecholamines, regulating energy mobilization, substrate availability, immune responses, and adaptation to physical stress [78].

Disruption of hormonal balance could theoretically influence recovery, body composition, training adaptation, and metabolic flexibility. For example, impaired androgen signaling could affect muscle remodeling, altered cortisol regulation could influence stress recovery, and disruption of thyroid hormone signaling could affect resting metabolic rate and substrate use. However, these possibilities stay hypothetical in relation to MPs. No study has directly proven that dietary MP exposure alters anabolic–catabolic balance, endocrine adaptation to exercise, or muscle protein synthesis in athletes.

Therefore, endocrine implications in athletes should be framed as a biologically plausible research question rather than as an established performance-limiting mechanism.

### 7.4. Metabolic and Reproductive Endocrine Pathways

Endocrine disruption may also influence energy metabolism. Hormonal signaling regulates nutrient partitioning between storage and oxidation, insulin sensitivity, glycogen storage, lipid mobilization, and appetite-related pathways. Experimental studies suggest that MPs and associated EDCs may alter glucose and lipid metabolism through effects on insulin signaling, hepatic metabolism, adipose tissue function, inflammatory pathways, and microbiota-related mechanisms [15,50,74,77,79]. However, the magnitude of these effects under realistic human dietary exposure remains unclear.

Reproductive endocrine pathways may also be relevant. Recent toxicological reviews have highlighted possible effects of micro- and nanoplastics on reproductive health, including steroidogenesis, gonadal function, oxidative stress, and hormone receptor signaling [43]. These mechanisms may be relevant to general human health, but their implications for athletes have not been specifically investigated. In female athletes, endocrine balance is particularly important for menstrual function, energy availability, bone health, and adaptation to training; in male athletes, androgen signaling is relevant to muscle remodeling and recovery. Nevertheless, no direct evidence currently links MP exposure to these outcomes in athletic populations.

### 7.5. Gut–Microbiota–Endocrine Interactions

An added layer of complexity involves interactions among MPs, the gut microbiota, and endocrine regulation. The gut microbiota contributes to the metabolism of hormones and hormone-like compounds and may influence enteroendocrine signaling, bile acid metabolism, appetite regulation, insulin sensitivity, and systemic inflammation [80,81,82,90,91]. As discussed previously, MP-induced dysbiosis may theoretically affect endocrine function indirectly by altering microbial metabolites, short-chain fatty acid production, gut barrier integrity, and inflammatory signaling [5,12,19,53]. This gut–microbiota–endocrine framework may be relevant for athletes because exercise, diet, energy availability, and gut microbiota are closely interconnected. However, the pathway linking dietary MP exposure, microbiota disruption, endocrine regulation, and performance-related outcomes stays untested. At present, this should be interpreted as a hypothesis-generating mechanism.

### 7.6. Endocrine Disruption: Limitations and Future Directions

It is important to note that most evidence about MP-related endocrine disruption derives from in vitro studies, animal models, and toxicological reviews, while direct human data remain limited [13,15,81,82]. Moreover, many studies evaluate either plastic-associated chemicals or defined experimental particles, making it difficult to determine whether observed endocrine effects are attributable to MPs themselves, associated EDCs, or combined exposures.

The endocrine system may be sensitive to low-dose and chronic exposures, particularly in the context of endocrine-disrupting chemicals; however, the magnitude, timing, and real-world relevance of MP-associated endocrine effects in humans remain uncertain [5,12,43,75]. These uncertainties are amplified in athletes, where hormonal responses are strongly influenced by sex, age, training status, energy availability, sleep, psychological stress, competition phase, and dietary intake [78,92].

Future research should investigate the relationship between dietary MP exposure, internal MP burden, EDC biomarkers, circulating hormone concentrations, menstrual function, anabolic–catabolic balance, thyroid function, insulin sensitivity, and recovery-related outcomes in athletes. Reliable biomarkers of MP exposure and endocrine disruption would be particularly valuable for improving exposure assessment and risk evaluation [11,45,50].

Overall, MPs may contribute to endocrine disruption through the transport and release of hormone-active compounds and possibly through indirect effects involving oxidative stress, inflammation, metabolism, and the gut microbiota [12,13,15,43,93,94]. While these mechanisms are biologically plausible, their relevance for endocrine regulation, muscle adaptation, recovery, and performance-related outcomes in athletes remains to be proven. Current conclusions should therefore be interpreted cautiously and should not be considered evidence of a demonstrated endocrine effect in athletic populations.

## 8. Implications for Sports Nutrition

### 8.1. Interpretation of the Evidence

Dietary microplastic exposure may plausibly have implications for sports nutrition through potential interactions with gastrointestinal function, gut microbiota composition, oxidative and inflammatory balance, mitochondrial function, endocrine regulation, and metabolic homeostasis [2,5,11,13,19,22]. However, these implications remain largely indirect. Direct evidence linking dietary MP exposure to nutrient absorption, recovery, training adaptation, or performance-related outcomes in athletes is currently lacking.

Therefore, the relevance of MPs for sports nutrition should be interpreted within a cautious framework. Current knowledge is mainly derived from food contamination studies, exposure-estimation models, in vitro experiments, animal studies, and indirect human evidence [4,11,12]. These data support biological plausibility, but they do not yet justify MP-specific sports nutrition guidelines.

### 8.2. Gastrointestinal Function and Nutrient Availability

Efficient gastrointestinal function is essential for athletes because energy availability, glycogen restoration, amino acid delivery, micronutrient status, hydration, and recovery depend on adequate digestion and absorption [95]. As discussed in earlier sections, experimental evidence suggests that MPs may interact with the intestinal epithelium, mucus layer, gut barrier, and microbiota [19,47,53,79]. These mechanisms could theoretically influence nutrient handling, especially under conditions of gastrointestinal stress. This aspect may be relevant in endurance and ultra-endurance sports, where exercise-induced gastrointestinal symptoms, altered permeability, and high intake of fluids or sports nutrition products often coexist [48,95]. Nevertheless, there is currently no direct evidence that MPs reduce carbohydrate, amino acid, lipid, vitamin, or mineral absorption in athletes. Therefore, any relationship between MP exposure and nutrient availability should be considered hypothetical.

### 8.3. Energy Metabolism, Recovery, and Adaptation

Potential MP-related effects on oxidative stress, inflammation, mitochondrial function, glucose metabolism, lipid metabolism, and endocrine signaling may be relevant to sports nutrition because these pathways contribute to energy production, substrate utilization, tissue repair, immune regulation, and training adaptation [15,67,68,96,97]. However, these mechanisms have been described mainly in preclinical models and should not be interpreted as evidence that MPs impair athletic performance.

From a practical perspective, sports nutrition principles are still the priority. Athletes should support adequate energy availability, carbohydrate intake proper to training load, sufficient protein intake for muscle remodeling, proper hydration, and correction of micronutrient deficiencies when present [95,98]. At present, there is no evidence that these recommendations should be changed specifically because of MP exposure.

### 8.4. Hydration and Sports Nutrition Products as Possible Exposure Sources

Hydration strategies deserve specific consideration because bottled water and packaged beverages have been repeatedly identified as potential sources of MP exposure [4,7,11,26,27,28]. Athletes may consume large volumes of water, sports drinks, recovery beverages, and energy drinks during training, competition, and travel. This may increase opportunities for exposure through plastic bottles, caps, flexible containers, and repeated use of sports bottles.

Similarly, sports nutrition products such as protein powders, carbohydrate supplements, energy bars, gels, meal replacements, and ergogenic aids are often packaged in plastic materials and may be repeatedly handled, scooped, shaken, or stored in plastic containers [31,32,99]. However, direct quantity of MPs in sports supplements is still limited. Therefore, these products should be viewed as plausible exposure sources rather than proved major contributors to internal MP burden in athletes.

### 8.5. Practical Strategies: Evidence-Based Nutrition Versus Precautionary Exposure Reduction

Practical recommendations should distinguish between established sports nutrition strategies and precautionary measures aimed at reducing avoidable MP exposure. Evidence-based sports nutrition should continue to focus on energy balance, nutrient timing, carbohydrate availability, protein distribution, hydration, micronutrient adequacy, and recovery support [95,98]. By contrast, MP-related strategies should currently be considered precautionary. These may include reducing unnecessary reliance on single-use plastic packaging, avoiding heating foods or beverages in plastic containers, limiting prolonged storage of drinks in plastic bottles under heat or sunlight, using filtered water when possible, and preferring glass or stainless-steel containers for repeated use [4,11,12]. Such measures may reduce avoidable exposure without compromising hydration, fueling, or recovery strategies. Importantly, exposure reduction should not lead to nutritionally inappropriate restrictions. Foods that may contribute to MP exposure, including seafood, fruits, vegetables, and packaged sports products, may also provide relevant nutritional benefits [2,9,14,33]. Therefore, the aim should not be to eliminate these foods, but to improve food quality, packaging choices, and preparation practices within a balanced risk–benefit framework.

### 8.6. Nutritional Resilience Strategies

Several dietary strategies may support physiological resilience to oxidative, inflammatory, gastrointestinal, and metabolic stress. Diets rich in fruits, vegetables, whole grains, legumes, nuts, extra-virgin olive oil, and fish provide polyphenols, carotenoids, omega-3 fatty acids, dietary fiber, antioxidant micronutrients, and other bioactive compounds that may support redox balance, inflammatory regulation, gut barrier integrity, and microbiota diversity [95,100,101,102,103,104,105]. Mediterranean-style dietary patterns are particularly relevant in this context because they combine high nutrient density with anti-inflammatory and antioxidant properties. Adequate dietary fiber and fermented or probiotic-containing foods may also support gut microbial diversity, short-chain fatty acid production, and intestinal barrier function [56,59,64,90]. These strategies are consistent with general health and sports nutrition recommendations. However, they should not be presented as proven interventions against MP toxicity. At present, there is no direct evidence that polyphenols, omega-3 fatty acids, probiotics, or antioxidant-rich diets prevent MP absorption, enhance MP elimination, or reverse MP-related biological effects in athletes.

### 8.7. Future Directions for Sports Nutrition Research

Future studies should evaluate MP exposure within real-world sports nutrition contexts, considering current methodological limitations in exposure assessment, particle detection, and human biomonitoring [4,11,12,44,45]. Priority areas include the assessment of MP contamination in sports drinks, protein powders, gels, bars, dietary supplements, shaker bottles, reusable sports containers, and packaged recovery products [7,14,26,27,28,31,32,99]. Human studies should combine dietary exposure assessment with biomarkers of internal MP burden, gastrointestinal permeability, microbiota composition, oxidative stress, inflammation, endocrine function, nutrient status, and recovery-related outcomes [5,11,12,15,36,42]. In athletes, these outcomes should be interpreted alongside training load, energy availability, hydration practices, supplement use, gastrointestinal symptoms, sleep, sex, and competition phase, all of which may influence physiological responses to environmental and nutritional stressors [92,95,98]. Intervention studies comparing highly packaged versus minimally packaged sports nutrition strategies could help determine whether modifiable dietary practices influence MP exposure. However, such studies should also ensure that performance-relevant nutritional targets are supported, including energy intake, carbohydrate availability, hydration, protein adequacy, micronutrient status, and recovery support [95,98].

Overall, MPs may plausibly intersect with sports nutrition through gastrointestinal, microbial, metabolic, endocrine, oxidative, and inflammatory pathways [5,12,13,15]. However, current implications remain largely theoretical and hypothesis-generating. Until direct athlete-specific evidence becomes available, practical guidance should prioritize established sports nutrition principles while adopting reasonable, low-risk measures to reduce avoidable plastic-related exposure.

## 9. Potential Implications for Recovery, Adaptation, and Performance-Related Outcomes

### 9.1. Evidence Framework and Interpretation

The possible implications of microplastic exposure for athletic performance, recovery, and training adaptation remain largely theoretical. Direct evidence linking dietary or environmental MP exposure to performance-related outcomes in athletes is currently lacking. Therefore, the available literature should be interpreted as an indirect evidence framework based on food contamination studies, human biomonitoring, in vitro experiments, animal models, and mechanistic toxicology [4,5,11,12,15]. Several biological pathways discussed in previous sections may be relevant to exercise-related physiology, including oxidative stress, inflammatory signaling, mitochondrial perturbation, gut barrier dysfunction, microbiota alterations, endocrine disruption, and metabolic dysregulation [13,15,21,22,54,65,66]. However, these mechanisms have not been directly confirmed in athletes. Thus, the potential implications described below should be considered hypothesis-generating rather than evidence of established effects on performance, recovery, or adaptation.

Table 2 summarizes the level of evidence linking MP exposure to selected sport-relevant biological domains. The table is intended to clarify the distinction between direct athlete-specific evidence, indirect human evidence, and mechanistic or preclinical findings.

### 9.2. Bioenergetics, Fatigue, and Mitochondrial Function

One potential pathway involves mitochondrial function and energy metabolism. Experimental studies suggest that MPs and nanoplastics may contribute to oxidative stress, alter mitochondrial membrane potential, impaired oxidative phosphorylation, and reduced ATP production [22,65,71,72,73,106]. In principle, such alterations could be relevant to exercise physiology, because endurance capacity, repeated high-intensity efforts, and recovery depend on efficient mitochondrial function and substrate oxidation.

However, the translation of these findings to athletes stays uncertain. Most data derive from cell and animal models, often using defined particles, selected polymers, and exposure conditions that may not reflect real-world dietary exposure. Some animal studies have reported reduced endurance-related outcomes following MP exposure [73], but these findings cannot be directly extrapolated to human athletes. Therefore, MP-related mitochondrial dysfunction should be considered a plausible mechanistic pathway, not an established cause of fatigue or reduced performance.

Fatigue is a multifactorial phenomenon involving metabolic, neuromuscular, inflammatory, and central nervous system components. MP-associated oxidative stress and inflammation could theoretically contribute to fatigue-related mechanisms [22,66,110]. At the muscular level, excessive ROS may affect excitation–contraction coupling and mitochondrial efficiency, while systemic inflammation may influence substrate availability, perceived exertion, and recovery. Nevertheless, direct evidence in athletic populations is lacking.

### 9.3. Respiratory Exposure and Oxygen-Related Performance Determinants

Oxygen uptake, transport, and use are central determinants of endurance performance. MPs may be relevant not only through dietary exposure, but also through inhalation, particularly in athletes training in urban environments, indoor facilities, or synthetic-surface settings [3,6,17,25,107]. Airborne MPs and associated pollutants may interact with the respiratory tract and contribute to local irritation or inflammatory responses, although the extent and functional relevance of these effects in athletes remain unknown. Given the importance of maximal oxygen uptake and pulmonary function for endurance performance [111], it is reasonable to consider respiratory MP exposure as a research priority. However, there is currently no direct evidence showing that airborne MPs impair VO_2_max, gas exchange, respiratory tolerance, or endurance capacity in athletes. Future studies should distinguish dietary exposure from inhalation exposure and evaluate sport-specific environments, including indoor training facilities, urban road training, and synthetic turf fields.

### 9.4. Recovery, Muscle Adaptation, and Training Tolerance

Efficient recovery depends on the coordinated restoration of energy stores, resolution of inflammation, repair of damaged tissues, mitochondrial remodeling, and protein turnover. MP-associated oxidative stress, inflammatory activation, endocrine disruption, and mitochondrial perturbation could theoretically interfere with these processes [13,15,21,22,66,106,110]. However, these links are still indirect and have not been tested in athletic populations. Muscle adaptation is another potential area of interest. Experimental evidence suggests that MPs may affect intracellular signaling pathways related to protein homeostasis, including PI3K/Akt/mTOR-related pathways [106,108,109]. Because these pathways are involved in muscle protein synthesis and adaptive remodeling, they may be relevant to resistance training and recovery. Nevertheless, no study has proved that MP exposure impairs muscle protein synthesis, hypertrophy, strength development, or training adaptation in athletes.

Therefore, statements about muscle adaptation should remain cautious. The current evidence supports biological plausibility, but not a direct performance-related effect.

### 9.5. Gastrointestinal Tolerance and Fueling During Exercise

The interaction between MP exposure and exercise-induced gastrointestinal disturbances may be relevant for sports nutrition. Gastrointestinal symptoms are common in endurance and ultra-endurance athletes and may impair fueling, hydration, and performance during prolonged exercise [48,112]. As discussed in earlier sections, experimental evidence suggests that MPs may interact with intestinal barrier function, mucus secretion, gut microbiota, and inflammatory signaling [19,46,47,53,63,79]. This creates a plausible but untested scenario in which MP exposure could interact with exercise-induced gastrointestinal stress. Such interaction might theoretically influence nutrient tolerance, carbohydrate availability, hydration strategies, and post-exercise recovery. However, no study has directly evaluated MP exposure, gastrointestinal permeability, GI symptoms, and exercise outcomes in athletes. This should therefore be considered a priority for future research rather than a current basis for practice recommendations.

### 9.6. Chronic Exposure and the Athletic Exposome

MP exposure is likely to be chronic, low-dose, and multi-source, involving food, beverages, air, food-contact materials, and sport-specific environments [2,3,6,8,10,14]. In athletes, this exposure may occur alongside other physiological and environmental stressors, including repeated training load, heat stress, air pollution, travel, sleep disruption, psychological stress, and high supplement use. This supports the concept of an athletic “exposome”, in which multiple external and internal exposures may interact with training adaptation and health outcomes [113].

At present, the contribution of MPs to the athletic exposome is still unknown. However, this framework may be useful for future studies because it avoids considering MPs as isolated exposures and instead situates them within the broader context of sports nutrition, environmental exposure, recovery, and physiological stress.

### 9.7. Research Priorities

Future research should prioritize controlled human studies evaluating MP exposure in relation to performance-related physiology rather than performance alone. Relevant outcomes include endurance capacity, strength, repeated-sprint ability, recovery kinetics, gastrointestinal tolerance, fatigue-related biomarkers, oxidative stress, inflammation, mitochondrial function, endocrine status, nutrient availability, and microbiota composition [5,11,12,95,98]. Studies should also include reliable exposure assessment methods, biomonitoring matrices, and standardized analytical protocols to distinguish external contamination from internal exposure [4,44,45]. In athletes, exposure should be evaluated alongside training load, dietary intake, hydration practices, supplement use, sex, age, energy availability, sleep, and competition phase. Intervention studies comparing highly packaged versus minimally packaged sports nutrition strategies may help determine whether modifiable dietary practices reduce MP exposure without compromising fueling, hydration, or recovery [7,14,26,27,28,31,32,95,98,99]. Environmental studies should also assess airborne MP exposure in indoor sport facilities, urban training environments, and synthetic-surface fields, where inhalation exposure and particle resuspension may be relevant [3,6,17,25,38,107].

**Figure 3:**
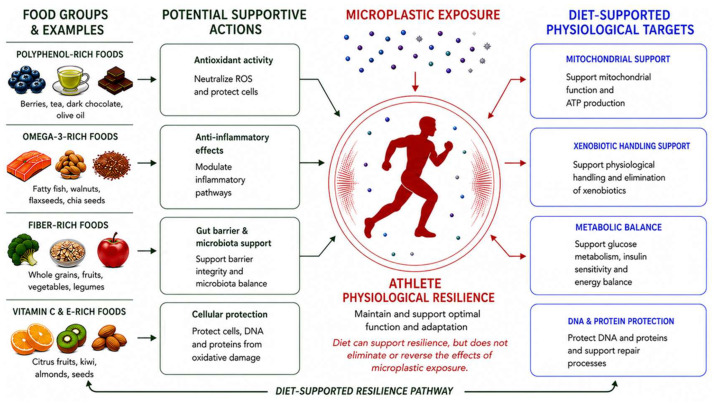
Evidence-informed nutritional strategies to support physiological resilience in athletes exposed to microplastics. Nutrient-rich dietary patterns may support physiological resilience through antioxidant activity, anti-inflammatory effects, gut barrier and microbiota support, and cellular protection. These actions may contribute to diet-supported physiological targets, including mitochondrial function, xenobiotic handling, metabolic balance, and DNA/protein protection. The figure should be interpreted as a conceptual framework for supportive sports nutrition strategies, not as evidence that diet can eliminate microplastic exposure or reverse microplastic-related biological effects.

Overall, current data support the existence of biologically plausible pathways through which MPs may intersect with performance-related physiology, including oxidative stress, inflammatory activation, mitochondrial dysfunction, gut barrier disruption, microbiota alterations, endocrine disruption, and metabolic dysregulation [5,11,12,13,15]. However, direct evidence in athletes is absent, and causal conclusions cannot be drawn. Until human studies become available, MP-related implications for performance, recovery, and adaptation should be interpreted cautiously as hypotheses for future research rather than as established effects (Figure 3).

## 10. Evidence-Informed Nutritional Strategies

### 10.1. General Framework

Given the current uncertainty surrounding microplastic exposure and its biological relevance in athletes, nutritional strategies should be framed as precautionary and supportive rather than therapeutic or prescriptive. Available evidence suggests that dietary exposure to MPs may occur through contaminated foods, bottled water, packaged beverages, food-contact materials, and processed products [2,4,8,10,11,12,14,26]. However, direct evidence showing that specific dietary interventions reduce internal MP burden or improve MP-related biological outcomes in athletes is currently lacking.

Therefore, practical approaches should adopt a dual strategy: reducing avoidable exposure where feasible and supporting physiological resilience through established principles of sports nutrition. This approach should not replace evidence-based recommendations for energy availability, carbohydrate intake, protein distribution, hydration, micronutrient adequacy, and recovery support [95,98]. Rather, MP-aware strategies should be integrated cautiously into existing nutrition practices without compromising performance, recovery, or dietary quality.

### 10.2. Reducing Avoidable Dietary Exposure

The first practical approach involves reducing avoidable contact with plastic materials during food selection, storage, preparation, and consumption. Prioritizing fresh and minimally processed foods may help limit exposure to packaging-derived particles and additives, although contamination can also occur in unprocessed foods through environmental and food-chain pathways [8,14,114]. Therefore, minimally processed diets should be recommended primarily because of their established nutritional value, with MP exposure reduction considered a potential additional benefit. Reducing unnecessary reliance on single-use plastic packaging, avoiding prolonged storage of foods and beverages in plastic containers, and preferring glass or stainless-steel containers when feasible may represent low-risk precautionary measures [4,11,12]. Heating foods in plastic containers should also be avoided where possible, as heat may promote the release of plastic particles and associated additives from food-contact materials [1,3,11].

Hydration practices are particularly relevant for athletes because fluid intake may be high during training, competition, heat exposure, and recovery. Bottled water and packaged beverages have been repeatedly identified as potential contributors to dietary MP exposure [4,7,11,26,27,28]. Supporting adequate hydration is still essential; however, when feasible, filtered tap water and non-plastic reusable containers may help reduce avoidable exposure without compromising hydration strategies [95].

### 10.3. Sports Nutrition Products and Supplements

Sports nutrition products require specific consideration because athletes often use protein powders, carbohydrate supplements, gels, bars, meal replacements, recovery drinks, and ergogenic aids. These products are often packaged in plastic containers or multilayer materials and may be repeatedly handled, scooped, mixed, shaken, or stored before consumption [24,25,75]. Such practices may be plausible exposure pathways, although direct quantification of MPs in sports supplements remains limited.

At present, there is no evidence that athletes should discontinue evidence-based supplements solely because of potential MP exposure. Instead, supplement use should remain guided by need, efficacy, safety, quality control, and sport-specific relevance [95,98]. From a precautionary perspective, athletes may reduce unnecessary supplement use, choose products from manufacturers with transparent quality-control procedures, avoid excessive storage in warm environments, and minimize repeated use of degraded plastic shakers or containers.

### 10.4. Supporting Antioxidant and Anti-Inflammatory Resilience

In addition to exposure reduction, dietary strategies may support physiological resilience to oxidative, inflammatory, gastrointestinal, and metabolic stress. Experimental evidence suggests that MPs may contribute to oxidative stress and inflammatory activation, although these effects have not been directly demonstrated in athletes [5,12,13,15,20,22,65,66]. Therefore, antioxidant- and anti-inflammatory dietary approaches should be presented as general resilience-supporting strategies, not as proven countermeasures against MP toxicity.

Diets rich in fruits, vegetables, whole grains, legumes, nuts, extra-virgin olive oil, herbs, and other plant-derived foods provide vitamin C, vitamin E, carotenoids, polyphenols, fiber, and other bioactive compounds that support redox balance, inflammatory regulation, endothelial function, and recovery [100,101,102,103,104,105,115,116,117,118,119,120]. Polyphenol-rich foods such as berries, tea, cocoa, grapes, and olive oil may be particularly relevant because of their antioxidant and anti-inflammatory properties [116,117,118,119,120]. However, current evidence does not show that polyphenols prevent MP absorption or reverse MP-induced effects in athletes. Omega-3 fatty acids, particularly eicosapentaenoic acid and docosahexaenoic acid, may support inflammatory balance, membrane function, and recovery-related processes [121,122]. Fish remains an important source of omega-3 fatty acids and high-quality protein, but seafood can also contribute to MP exposure through food-chain contamination [2,9,33]. Therefore, recommendations should be based on a balanced risk–benefit perspective rather than avoidance. Algae-based omega-3 supplements may be considered in selected cases, especially when dietary intake is insufficient or when athletes prefer non-fish sources.

### 10.5. Supporting Gut Health and Microbiota Resilience

Gut health is another relevant target because gut microbiota contributes to nutrient metabolism, short-chain fatty acid production, immune regulation, intestinal barrier integrity, and exercise-related physiological adaptation [50,51,59,64]. Experimental studies suggest that MP exposure may alter microbiota composition and intestinal homeostasis in animal models, but direct human and athlete-specific evidence remains limited [5,19,50,53,55].

Adequate dietary fiber intake from vegetables, fruits, legumes, whole grains, nuts, and seeds may support microbial diversity and SCFA production [48,56,59]. Fermented foods and probiotic-containing products may also support microbiota balance in selected contexts, although evidence specific to MP exposure is not available [123]. Prebiotic compounds may further contribute to microbial resilience. These strategies are consistent with general sports nutrition and gut-health recommendations, but they should not be presented as MP-specific treatments.

### 10.6. Balanced Risk–Benefit Interpretation

All proposed strategies should be interpreted within a balanced risk–benefit framework. Many foods and products that may contribute to MP exposure, including seafood, fruits, vegetables, bottled beverages, and sports nutrition products, may also provide important nutritional or practical benefits [2,9,14,33,95,121]. Therefore, the goal should not be to eliminate these foods or products, but to improve their use while reducing unnecessary plastic-related exposure. This distinction is particularly important in athletes. Excessive restriction, inadequate fueling, poor hydration, or avoidance of useful sports nutrition products may cause greater harm than the uncertain risk associated with MP exposure. Thus, MP-aware strategies should be practical, low-risk, and compatible with adequate energy intake, nutrient timing, hydration, recovery, and training adaptation [95,98].

Overall, evidence-informed nutritional strategies should combine reasonable exposure reduction with support for physiological resilience. Athletes may benefit from dietary patterns rich in minimally processed, nutrient-dense foods; adequate hydration practices; proper use of evidence-based supplements; and nutritional strategies that support antioxidant defenses, inflammatory balance, gut health, and recovery. However, these approaches should be considered precautionary and supportive rather than proven interventions against MP-related biological effects.

## 11. Limitations of Current Evidence and Future Perspectives

A central limitation of the current literature is the indirect nature of the evidence linking microplastic exposure to sports nutrition, recovery, adaptation, and performance-related outcomes. At present, most proposed implications are based on biological plausibility, food contamination studies, exposure-estimation models, in vitro experiments, animal studies, and indirect evidence from non-athletic human populations [4,5,11,12,15]. Direct intervention studies, longitudinal analyses, and biomonitoring investigations specifically conducted in athletes are currently lacking.

Most mechanistic evidence derives from preclinical models. These studies are useful for identifying potential pathways, including oxidative stress, inflammatory activation, mitochondrial perturbation, gut barrier dysfunction, microbiota alterations, endocrine disruption, and metabolic dysregulation [5,12,13,15]. However, experimental models often use selected polymer types, defined particle sizes, controlled exposure conditions, and doses that may not reflect chronic low-dose dietary exposure in humans. Therefore, these findings should not be directly interpreted as evidence of clinically meaningful effects on athletic performance or recovery.

Another important limitation concerns the heterogeneity of MPs across studies. Particle size, shape, polymer composition, surface charge, aging, concentration, exposure duration, and the presence of adsorbed contaminants or plastic additives may all influence biological responses [4,11,12]. This variability complicates comparisons across studies and limits the generalizability of current findings. In addition, many studies do not clearly distinguish between the effects of plastic particles themselves and those mediated by associated chemicals, including endocrine-disrupting compounds, heavy metals, or persistent organic pollutants [3,13,43].

A further limitation is the lack of standardized analytical methods for detecting and quantifying MPs and nanoplastics in foods, beverages, environmental samples, and human biological matrices. Differences in sampling procedures, contamination-control protocols, digestion and extraction methods, polymer-identification techniques, particle-size detection limits, and reporting units reduce comparability among studies [3,11,44,45]. Although MPs have been detected in several human matrices, including blood, feces, placenta, lung tissue, liver tissue, atherosclerotic plaques, and skeletal tissues, these findings demonstrate detectability rather than absorption efficiency, dose–response relationships, tissue-specific toxicity, or functional impairment [16,36,37,38,42].

The absence of athlete-specific quantitative exposure data is a particularly relevant gap. Athletes may have distinctive exposure profiles because of high food and fluid intake, frequent use of packaged sports nutrition products, supplement consumption, travel, indoor training, urban exercise, and contact with synthetic sport surfaces [6,7,14,17,26,27,28,31,32,99]. However, no study has yet quantified internal MP burden in athletes or examined whether exposure differs according to sport discipline, training load, dietary pattern, hydration strategy, or supplement use. As a result, risk estimation in athletic populations is still highly uncertain.

Future research should prioritize well-designed human studies integrating dietary exposure assessment, environmental monitoring, validated biomonitoring approaches, and standardized analytical protocols [4,11,12,44,45]. In athletic populations, these studies should evaluate MP exposure in relation to sport-specific variables, including training load, dietary intake, hydration practices, supplement use, energy availability, sex, environmental conditions, recovery, and performance-related physiology [92,95,98].

Intervention studies comparing highly packaged and minimally packaged sports nutrition strategies may help decide whether modifiable dietary practices can reduce MP exposure without compromising fueling, hydration, recovery, or nutrient adequacy [7,14,26,27,28,31,32,95,98,99]. However, such approaches should be designed within a balanced risk–benefit framework to avoid inappropriate restriction of foods or products with established nutritional and performance benefits [2,9,33,95,121].

Overall, current evidence supports the biological plausibility of interactions between MP exposure and pathways relevant to sports nutrition and exercise physiology, including gastrointestinal, microbial, oxidative, inflammatory, mitochondrial, endocrine, and metabolic pathways [5,11,12,13,15]. However, direct evidence in athletes is absent, analytical methods stay insufficiently standardized, and causal relationships cannot be established [4,44,45]. Therefore, conclusions regarding MP exposure, recovery, adaptation, and performance-related outcomes should remain cautious and hypothesis-generating until supported by robust human and athlete-specific studies.

## 12. Conclusions

Microplastics represent an emerging issue in sports nutrition, with dietary exposure being a relevant route of human intake. Athletes may present distinctive exposure scenarios because of elevated food and fluid consumption, frequent use of packaged sports nutrition products, dietary supplements, and sport-specific hydration practices.

Current evidence suggests that MPs may interact with biological pathways relevant to gastrointestinal function, gut microbiota, oxidative stress, inflammation, mitochondrial activity, endocrine regulation, and metabolic homeostasis. However, most data derive from in vitro studies, animal models, and indirect human evidence. Direct studies in athletic populations are currently lacking.

Therefore, the possible implications of MP exposure for recovery, adaptation, and performance-related outcomes should be considered biologically plausible but unproven. These effects should not be interpreted as evidence that MPs impair athletic performance. Until athlete-specific data become available, practical guidance should remain precautionary and evidence-informed. Reasonable strategies may include reducing avoidable plastic-related exposure while supporting adequate hydration, energy intake, nutrient timing, supplement quality, and dietary patterns that support antioxidant defenses, inflammatory balance, and gut health.

Future research should prioritize standardized exposure assessment, validated biomarkers, human biomonitoring studies, and sport-specific investigations evaluating the relationship between MP exposure, physiological responses, recovery, and performance-related outcomes.

## Figures and Tables

**Table 1:** Representative evidence on microplastic exposure, biological effects, and relevance to athletes.

Evidence Area	Study Model/Population	Main Approach	Key Findings	Relevance and Limitations for Athletes	Representative References
Dietary exposure	General human population; food and beverage contamination studies	Exposure estimates based on contaminated food, water, beverages, and food-contact materials	Dietary intake is a relevant route of MP exposure, but estimates vary widely according to matrix, particle size, and analytical method	Relevant because athletes may have higher food and fluid intake; no athlete-specific exposure estimates are available	[2,4,10,11,14,47]
Gastrointestinal uptake and translocation	In vitro intestinal models and animal studies	Defined MPs/NPs; epithelial barrier models; tissue distribution analyses	Smaller MPs and NPs may interact with intestinal epithelium and, under experimental conditions, translocate beyond the gut	Supports biological plausibility, but evidence is mainly preclinical and often based on high-dose or controlled exposures	[5,12,13,15,34,35]
Human biomonitoring	Human blood, placenta, lung, liver, vascular, and skeletal tissues	Raman spectroscopy, μFTIR, pyrolysis–GC/MS, and related polymer-identification methods	MPs/NPs have been detected in several human biological matrices	Demonstrates detectability, not toxicity, dose–response, functional impairment, or relevance to athletic performance	[16,36,37,38,39,40,41,42]
Gut microbiota and intestinal homeostasis	Rodent and zebrafish models; mechanistic reviews	MP exposure with microbiota, intestinal, inflammatory, and metabolic assessments	MPs may alter microbial composition, intestinal inflammation, oxidative stress, and microbial metabolism	Relevant to gut tolerance, nutrient metabolism, immunity, and recovery, but human and athlete-specific evidence is lacking	[5,19,50,53,55]
Oxidative, inflammatory, and mitochondrial pathways	In vitro studies, animal models, and toxicological reviews	Cellular and tissue biomarkers of ROS, cytokines, mitochondrial function, ATP production, and oxidative damage	MPs/NPs may induce oxidative stress, inflammatory activation, and mitochondrial perturbation	Mechanistically relevant to fatigue, recovery, and adaptation, but not demonstrated in athletes	[5,12,13,15,22,65,66,67,68,69,70,71,72,73]
Endocrine and metabolic disruption	Animal studies and toxicological reviews	Assessment of EDCs, hormone-related pathways, glucose/lipid metabolism, and reproductive endpoints	MPs and associated chemicals may interact with endocrine and metabolic pathways	Potential relevance to anabolic–catabolic balance, substrate use, and adaptation; difficult to separate particle effects from additive effects	[43,74,75,76,77,78,79,80,81,82]
Analytical and translational limitations	Human biomonitoring and methodological reviews	Comparison of detection methods, extraction protocols, contamination control, and reporting metrics	Lack of standardized methods limits comparability across studies and interpretation of internal exposure	Essential limitations for athlete studies; validated biomarkers and harmonized protocols are needed	[4,11,12,44,45]

Note. This table summarizes representative evidence rather than providing an exhaustive systematic synthesis. Human biomonitoring studies indicate detectability of MPs/NPs in biological matrices but do not establish toxicity, dose–response relationships, functional impairment, or relevance to athletic performance. MP, microplastic; NP, nanoplastic; EDC, endocrine-disrupting chemical; μFTIR, micro-Fourier transform infrared spectroscopy; GC/MS, gas chromatography–mass spectrometry; ROS, reactive oxygen species; ATP, adenosine triphosphate.

**Table 2:** Evidence map summarizing potential links between microplastic exposure and sport-related outcomes.

Biological Domain	Direct Evidence in Athletes	Main Evidence Source	Key Biological Findings	Plausible Sport-Related Relevance	Interpretation	Representative References
Oxidative stress	Absent	In vitro and animal studies	Increased ROS production, altered antioxidant defenses, lipid peroxidation, and oxidative cellular damage	May be relevant to redox balance, fatigue susceptibility, recovery, and training adaptation	Mechanistically plausible, not demonstrated in athletes	[5,12,20,22,65,66,67,68]
Inflammatory activation	Absent	In vitro, animal, and indirect human evidence	Activation of inflammatory pathways, including NF-κB signaling and cytokine production	May theoretically influence tissue repair, immune balance, recovery, and adaptation to repeated exercise stress	Plausible but unconfirmed	[13,15,19,21,54,66,67,70]
Mitochondrial function	Absent	Preclinical models	Altered mitochondrial membrane potential, impaired oxidative phosphorylation, increased ROS generation, and reduced ATP production	May be relevant to bioenergetic efficiency, endurance capacity, and fatigue resistance	Preclinical evidence only	[5,12,22,65,69,71,72,73,106]
Gut barrier function	Absent	Experimental and indirect evidence	Altered epithelial integrity, mucus layer disruption, and increased intestinal permeability	May interact with exercise-induced gastrointestinal stress, GI symptoms, and nutrient tolerance	Hypothesis-generating	[12,19,46,48,63,79]
Gut microbiota	Absent	Animal models and mechanistic reviews	Changes in microbial diversity, SCFA-producing taxa, gut inflammation, and microbial metabolism	May affect gut tolerance, immune regulation, nutrient metabolism, and recovery	Indirect and unconfirmed in athletes	[5,19,47,50,53,55,56,64]
Endocrine regulation	Absent	EDC research, animal studies, and mechanistic reviews	Possible interaction with hormone-related pathways through additives, plasticizers, and adsorbed contaminants	May theoretically influence anabolic–catabolic balance, endocrine regulation, body composition, and adaptation	Plausible but not proven	[9,43,74,75,77,78,79,80,81,82]
Glucose and lipid metabolism	Absent	Animal studies and mechanistic evidence	Altered insulin signaling, hepatic lipid metabolism, adiposity, and metabolic homeostasis	May be relevant to substrate use, energy availability, and metabolic flexibility	Low–moderate preclinical evidence	[5,50,74,75,76,79,93]
Respiratory exposure	Absent	Environmental and indirect human evidence	Airborne MPs may interact with respiratory tissues and contribute to local inflammatory responses	May be relevant for athletes training in urban, indoor, or synthetic-surface environments	Research priority, not established effect	[3,6,17,25,38,107]
Muscle adaptation and recovery	Absent	Limited preclinical evidence	Possible effects on protein homeostasis, PI3K/Akt/mTOR-related signaling, oxidative stress, and inflammation	May theoretically influence muscle repair, hypertrophy, recovery kinetics, and adaptation to resistance training	Very limited evidence	[15,65,106,108,109]
Exercise performance	Absent	Animal and mechanistic evidence only	Some experimental models suggest effects on endurance-related outcomes, but translation to humans is uncertain	Potential relevance to endurance, fatigue resistance, and recovery is still speculative	No causal evidence in athletes	[17,72,73,106,110]

Note. Direct evidence refers to studies in athletes or physically trained populations. Indirect human evidence includes non-athletic human studies, biomonitoring data, or general exposure research. Mechanistic/preclinical evidence includes in vitro, animal, or controlled experimental models. Because athlete-specific evidence is absent, sport-related implications should be interpreted as biologically plausible hypotheses, not established causal effects. MP, microplastic; ROS, reactive oxygen species; SCFA, short-chain fatty acid; EDC, endocrine-disrupting chemical; ATP, adenosine triphosphate; NF-κB, nuclear factor kappa B; PI3K/Akt/mTOR, phosphoinositide 3-kinase/protein kinase B/mechanistic target of rapamycin.

## Data Availability

All data supporting the findings of this study are reported within the manuscript.

## Abbreviations

ATP	adenosine triphosphate
DHA	docosahexaenoic acid
EDCs	endocrine-disrupting chemicals
EPA	eicosapentaenoic acid
ERs	estrogen receptors
GI	gastrointestinal
IGF-1	insulin-like growth factor 1
LPS	lipopolysaccharide
MPs	microplastics
mTOR	mechanistic target of rapamycin
NF-κB	nuclear factor kappa B
ROS	reactive oxygen species
SCFAs	short-chain fatty acids
VO_2_max	maximal oxygen uptake
